# Enfortumab Vedotin in Advanced or Metastatic Urothelial Carcinoma: A Systematic Review of Efficacy, Safety, and Clinical Perspectives

**DOI:** 10.3390/cancers18142324

**Published:** 2026-07-18

**Authors:** Julia Piekarz, Natalia Picheta, Jakub Pobideł, Karolina Daniłowska, Natalia Gierulska, Katarzyna Szklener, Magdalena Skórzewska

**Affiliations:** 1 Student Academic Group, Department of Clinical Oncology and Chemotherapy, Medical University, 20-954 Lublin, Poland; 59750@umlub.edu.pl (N.P.); 58108@umlub.edu.pl (J.P.); 57664@umlub.edu.pl (K.D.); 61669@umlub.edu.pl (N.G.); 2 Department of Clinical Oncology and Chemotherapy, Medical University, 20-954 Lublin, Poland; magdalena.skorzewska@umlub.edu.pl

**Keywords:** antibody–drug conjugates, enfortumab vedotin, urothelial carcinoma, Metastatic Urothelial Cancer, EV

## Abstract

**Background:** Urothelial carcinoma (UC) is a significant clinical problem, especially in locally advanced and metastatic stages, where the prognosis remains poor despite advances in immunotherapy. Enfortumab vedotin (EV), an antibody–drug conjugate (ADC) targeting Nectin-4, extends beyond direct cytotoxicity by actively modulating the immunosuppressive tumor microenvironment (TME) in preclinical models, creating a strong rationale for combination strategies. This systematic review aimed to evaluate the clinical efficacy, safety, and hypothesized immune-modulating potential of EV in the treatment of UC, particularly in combination with immunotherapy. **Materials and Methods:** The review was conducted in accordance with the PRISMA 2020 guidelines. PubMed, Web of Science, and ClinicalTrials.gov databases were searched to identify interventional clinical studies published between 2020 and 2026. Studies evaluating EV used as monotherapy or in combination with immunotherapy in adult patients with UC were analyzed. ORR, PFS, OS, and the incidence of treatment-related adverse events were evaluated. Individual publications originating from the same clinical trial programs were treated as linked records and analyzed collectively. **Results:** The analysis was primarily based on key clinical trial programs, including EV-103, EV-301, and EV-302, along with their subgroup analyses, follow-up reports, and patient-reported outcomes. EV demonstrated significant clinical efficacy in the treatment of locally advanced UC (laUC). In the EV-301 study, EV therapy significantly prolonged overall survival compared to chemotherapy (12.88 vs. 8.97 months). In the EV-302 study, EV combination therapy with pembrolizumab showed a significant improvement in progression-free survival and overall survival compared to platinum-based chemotherapy. **Conclusions:** EV represents a significant therapeutic advance not only as a cytotoxic agent but as a potent immune modulator with clinical efficacy that may be enhanced by its biologically hypothesized immunomodulatory mechanisms in mUC. Its clinical development reflects a critical shift from later-line monotherapy toward earlier-line combination strategies designed to target both tumor cells and the microenvironment. Further studies are needed to better define its role in the context of alternative treatment approaches and across different stages of disease.

## Simple Summary

Advanced bladder cancer, or urothelial carcinoma, is an aggressive disease with historically low survival rates and limited treatment options once standard therapies fail. Enfortumab vedotin is a promising targeted therapy called an antibody–drug conjugate that delivers chemotherapy directly to cancer cells while boosting the body’s immune response. This systematic review evaluates how well this drug works, its safety profile, and its ability to modulate the immune system, particularly when combined with standard immunotherapy. By analyzing data from key clinical trials, this research highlights that enfortumab vedotin significantly improves patient survival and quality of life. These insights help the research and medical communities understand a major therapeutic shift toward more effective, personalized, and earlier-line cancer treatments, ultimately guiding clinical decision-making

## 1. Introduction

Urothelial carcinoma (UC) is the tenth most common malignant tumor in the world, with an estimated 614,000 new cases and 220,000 deaths annually [1]. This cancer, which originates in the bladder in over 90% of cases, is characterized by an aggressive clinical course, especially in advanced stages. Over the past three decades, platinum-based chemotherapy has remained the standard first-line treatment for patients with metastatic urothelial carcinoma (mUC). Despite initially high response rates, most patients experience rapid disease progression, and the prognosis at this stage remains highly unfavorable—the 5-year overall survival (OS) rate in the era before the introduction of immunotherapy was only about 5% [2].

Although highly immunogenic—characterized by elevated tumor mutational burden (TMB), abundant tumor-infiltrating lymphocytes (TILs), and robust PD-L1 expression conferring potential susceptibility to immune checkpoint inhibitors (ICIs)— UC features a heterogeneous, profoundly immunosuppressive tumor microenvironment (TME) that limits ICI monotherapy efficacy. Consequently, prevalent resistance to both classic chemotherapy and Programmed Cell Death Protein 1/Programmed Death-Ligand 1 (PD-1/PD-L1) inhibitors dictates an urgent clinical need for novel therapeutics capable of synergizing direct cytotoxicity with immune stimulation [3]. Notably, fibroblast growth factor receptor (FGFR) alterations frequently contribute to the inhibition of anti-tumor immunity within the tumor microenvironment, actively attenuating clinical responses to immune checkpoint inhibitors (ICIs) and conventional chemotherapy. Consequently, balancing the distinct efficacy and safety profiles of novel targeted agents, such as selective pan-FGFR inhibitors, alongside emerging combination strategies remains crucial for disrupting these immunosuppressive mechanisms and improving patient outcomes [4].

Antibody–drug conjugates (ADCs) utilize a “Trojan horse” strategy by coupling monoclonal antibody specificity with potent chemotherapeutic payloads, inducing direct cell cycle arrest and apoptosis. Beyond direct cytotoxicity, ADCs like enfortumab vedotin (EV) induce immunogenic cell death (ICD) in preclinical models, a process characterized by the cell-surface exposure of calreticulin (“eat-me” signals) and the extracellular release of adenosine triphosphate (ATP) and high mobility group box 1 (HMGB1) protein (“find-me” signals). These damage-associated molecular patterns (DAMPs) are hypothesized to stimulate dendritic cell maturation and enhance antigen cross-presentation to T-cells, thereby effectively bridging direct tumor cytotoxicity with the activation of adaptive antitumor immunity [5].

Representing a benchmark in this class, EV targets Nectin-4 (PVRL4)—a type I adhesion protein highly overexpressed in UC with minimal presence in healthy tissues. EV comprises a human IgG1 antibody attached via a cleavable linker to the microtubule inhibitor monomethyl auristatin E (MMAE). Following internalization, intracellular MMAE release drives both direct apoptosis and a “bystander” effect, diffusing to eradicate adjacent cancer cells regardless of their antigen expression. Furthermore, emerging translational evidence indicates that Nectin-4 may actively function within the tumor microenvironment by interacting with inhibitory receptors such as TIGIT on immune cells, suggesting that targeting this axis could fundamentally complement co-administered immune checkpoint inhibitors [6].

The clinical efficacy of EV has been confirmed in key clinical trials. The phase III EV-301 trial showed a significant increase in overall survival (12.88 vs. 8.97 months) in patients previously treated with platinum and immunotherapy [7]. Furthermore, recent reports from the EV-302 (KEYNOTE-A39) study, evaluating the combination of EV with pembrolizumab, provide compelling clinical evidence of profound therapeutic synergy. By demonstrating unprecedented improvements in OS and progression-free survival (PFS) compared to conventional chemotherapy, this combination has revolutionized the standard of first-line treatment, supporting the hypothesis that targeted cytotoxicity can successfully complement immune checkpoint inhibition [8].

EV fundamentally improves the mUC prognosis and provides a compelling rationale for its integration into current immuno-oncology strategies. Consequently, this systematic review evaluates the clinical efficacy, safety profile, and immune-modulating potential of EV in UC, highlighting the latest translational evidence bridging targeted cytotoxicity with anti-tumor immunity.

## 2. Materials and Methods

### 2.1. Study Designs

The systematic review was conducted in accordance with the Preferred Reporting Items for Systematic Reviews and Meta-Analyses (PRISMA) 2020 guidelines. The aim of this review is to comprehensively evaluate the clinical efficacy and safety of EV (Seagen Inc., Bothell, WA, USA) in UC, with a specific focus on the preclinical rationale underlying its hypothesized immune-modulating potential and its therapeutic synergy when combined with immunotherapy.

The analysis included prospective clinical trial publications evaluating EV in adult patients with UC, including randomized phase III trials, phase Ib/II studies, long-term follow-up reports, predefined subgroup analyses, and patient-reported outcome (PRO) analyses derived from the same parent trial programs.

The review protocol was registered in the International Prospective Register of Systematic Reviews (PROSPERO) under registration number CRD420261383667.

### 2.2. Eligibility Criteria and PICO Framework

The research focused on evaluating the efficacy and safety of EV-based therapies. The PICO model was used to structure and focus the literature review.

- Population: The target population consists of adult patients diagnosed with UC, regardless of gender.
- Intervention: Interventions included EV as monotherapy or in combination with immunotherapy (e.g., pembrolizumab).
- Comparison: Comparators included standard chemotherapy or other treatment regimens, where applicable.
- Outcome: The primary outcomes analyzed focused on the clinical efficacy and safety of EV therapy. Efficacy endpoints—mainly objective response rate (ORR), PFS, OS, duration of response (DoR), complete response (CR), partial response (PR), stable disease (SD), and progressive disease (PD)—were evaluated to gauge the clinical impact of EV-mediated immune modulation, either as monotherapy or in synergistic combination with immune checkpoint inhibitors. Safety was evaluated based on the incidence and severity of treatment-related adverse events (TRAEs).

Studies published in English between 2020 and 2025 were eligible for inclusion. The review considered prospective clinical trials (phase I–III) evaluating EV as monotherapy or in combination with immunotherapy in adult patients with locally advanced UC (laUC) or mUC.

In addition to primary trial reports, secondary publications derived from the same parent clinical trial programs—including predefined subgroup analyses, PROs, and extended follow-up reports—were also included when they provided additional clinically relevant efficacy or safety data. Multiple publications originating from the same trial were treated as linked records and synthesized under a single trial program.

Exclusion criteria comprised review articles, preclinical studies (animal or cellular models), conference abstracts or posters without complete data, and publications lacking relevant efficacy or safety outcomes related to EV therapy.

A summary of the inclusion and exclusion criteria based on the PICO framework is presented in Table 1.

### 2.3. Search and Selection Process

A systematic search of PubMed, Web of Science, and ClinicalTrials.gov was performed to identify relevant studies published between 1 January 2020, and 31 March 2026. The final search was conducted on 19 April 2026. Given that the search targeted major, well-established multi-center prospective trial programs, these primary databases provided complete coverage, and supplementary searches confirmed no additional unique trials would be retrieved from alternative databases.

The search strategy was optimized for each database by utilizing a comprehensive combination of keywords, text words, and subject headings. The exact search string implemented across the fields was structured as follows: (“Enfortumab Vedotin” OR “EV”) AND (“Urothelial Cancer” OR “Metastatic Urothelial Cancer” OR “Urothelial Carcinoma” OR “Bladder Cancer”) AND (“Antibody–drug conjugate” OR “ADC”).

To ensure completeness, a manual search of the reference lists of all eligible studies was also performed. Two researchers (Ju.P. and N.P.) independently screened titles, abstracts, and full-text articles using predefined eligibility criteria. Any discrepancies were resolved through discussion with two additional reviewers (Ja.P. and K.D.).

ClinicalTrials.gov records were cross-checked against peer-reviewed publications to avoid duplicate inclusion of registry entries and published reports. When multiple publications originated from the same parent clinical trial, these were treated as linked records and synthesized under a single trial program.

The database search identified 1232 records. Prior to screening, 446 duplicate records and 98 records published before 2020 were removed (identified and removed using Zotero v7.0, Corporation for Digital Scholarship, Vienna, VA, USA) (totaling 544 records). Of the remaining 688 records subjected to title screening, 510 were excluded. Subsequently, 178 abstracts were screened, resulting in the exclusion of 122 records. A total of 56 full-text reports were assessed for eligibility, of which 47 were excluded (23 review articles, 13 in vitro studies, and 11 due to an irrelevant population or intervention). Ultimately, nine eligible publications representing three unique clinical trial programs were included in the final qualitative synthesis.

A formal quantitative meta-analysis was deemed methodologically inappropriate due to the substantial clinical heterogeneity across the included programs, which spanned distinct clinical phases (Phase Ib/II vs. Phase III) and fundamentally different treatment settings (EV monotherapy vs. EV combined with pembrolizumab).

**Figure 1:**
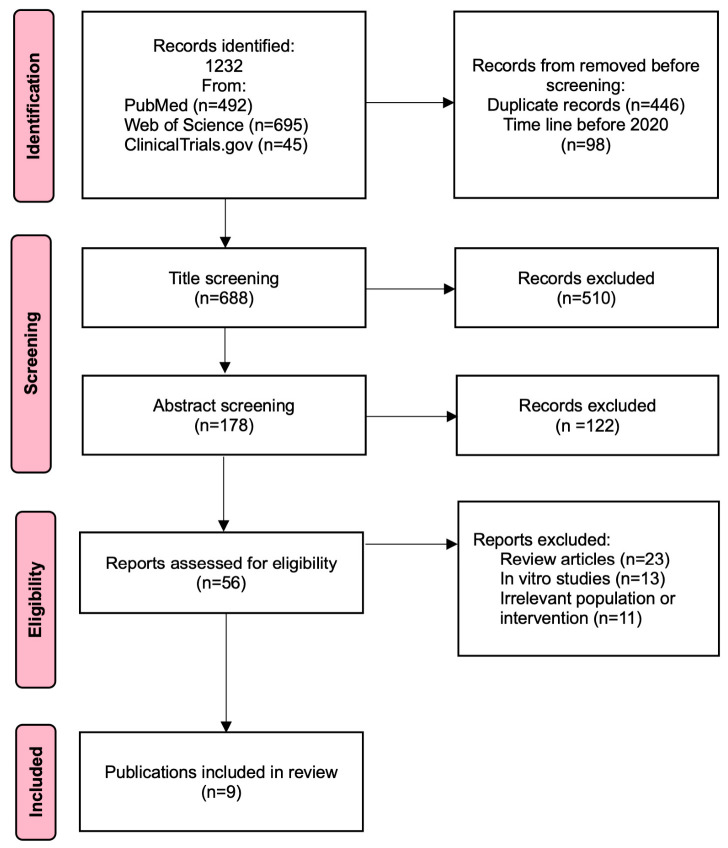
Preferred Reporting Items for Systematic Reviews and Meta-analyses (PRISMA). flow diagram of study identification, inclusion, and exclusion.

The study selection process, including identification, screening, eligibility, and inclusion, is presented in the PRISMA flow diagram in Figure 1.

### 2.4. Data Extraction

The review included nine publications derived from three major clinical trial programs (EV-103, EV-301, and EV-302/KEYNOTE-A39). These publications collectively reported data from 1643 unique patients, with linked secondary reports providing additional subgroup, long-term follow-up, and PRO data.

Data extraction was performed independently by two reviewers using a standardized form, with any discrepancies resolved through discussion or consultation with a third reviewer. Extracted variables included parent trial program, publication type (primary efficacy, subgroup, follow-up, or PRO report), study population, treatment regimen, and efficacy and safety outcomes. Specifically, extracted data encompassed study characteristics (author, year, study design), patient demographics (total number of patients, age, gender), intervention details (EV dosage, regimen, monotherapy vs. combination therapy), and outcome measures related to efficacy (ORR, PFS, OS, DoR, CR, PR, SD, PD, Clinical Benefit [CB]) and safety (incidence and severity of TRAEs).

### 2.5. Assessment of Risk of Bias in the Included Studies

**Figure 2:**
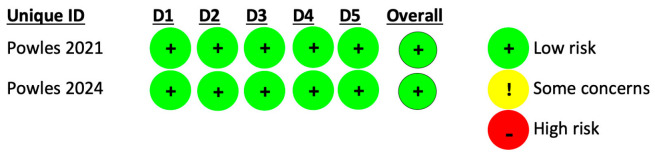
Risk of bias assessment for the included RCT using the Cochrane Risk of Bias 2.0 (RoB 2.0) tool. Risk of bias is evaluated across five specific domains: **D1**: Bias arising from the randomization process; **D2**: Bias due to deviations from the intended interventions; **D3**: Bias due to missing outcome data; **D4**: Bias in measurement of the outcome; and **D5**: Bias in selection of the reported result. The green circles with a plus sign (+) indicate a low risk of bias.

Risk of bias was assessed at the level of the parent randomized trials (RCTs) using the updated Cochrane Risk of Bias tool for randomized trials (RoB 2.0) (Higgins, 2019). This tool evaluates five domains: (1) bias arising from the randomization process, (2) bias due to deviations from intended interventions, (3) bias related to missing outcome data, (4) bias in outcome measurement, and (5) bias in the selection of reported results. Two independent reviewers applied RoB 2.0 to all parent trials, recording supporting evidence and providing judgments of low risk, high risk, or some concerns for each domain. Discrepancies were resolved through discussion or adjudication by a third reviewer. For linked secondary publications (such as subgroup, PRO, and long-term follow-up reports), risk-of-bias judgments were anchored to the corresponding parent trial, with additional consideration of reporting completeness and outcome-specific limitations. The domain-level RoB 2.0 summary for all parent randomized studies included in the review is shown in Figure 2.

For the single-arm cohorts or non-randomized evidence included in this review (O’Donnell et al., 2023), the methodological quality was evaluated using the NIH Quality Assessment Tool for Before-After (Pre-Post) Studies with No Control Group, following the same rigorous approach applied to other non-RCT designs. This study demonstrated high methodological rigor by clearly defining the study population, ensuring consistency in the intervention protocol (EV with or without Pembrolizumab), and utilizing standardized, pre-specified criteria for outcome tracking. Consistent with the inherent limitations of open-label, early-phase oncology trial designs, the primary domain where the study fell short was the blinding of outcome assessors. Despite this lack of blinding, the study was graded as having an overall quality rating of “Good,” indicating a low risk of bias and a high reliability of the reported clinical outcomes, such as objective response rates and survival metrics.

## 3. Urothelial Carcinoma

UC, formerly known as transitional cell carcinoma, is the most common type of urinary tract cancer, accounting for over 90% of all bladder cancer cases [9]. UC develops across the urothelium and encompasses typical histology alongside higher-grade morphological subtypes—such as squamous cell carcinoma (approx. 3%), adenocarcinoma (2%), and small cell carcinoma (<1%)—that correlate with poorer responses to standard therapies [10]. Ranking as the ninth most common global malignancy—with approximately 614,000 new cases and 220,000 deaths worldwide according to GLOBOCAN 2022 data, and recent 2026 estimates for the United States alone projecting 82,140 new cases and 16,540 deaths [1,11]. UC exhibits a strong male predilection (4:1 ratio; median age 73) and is heavily driven by tobacco smoking, which quadruples disease risk and accounts for 50% of cases, alongside occupational exposure to aromatic amines, radiation, and chronic inflammation [12]. Although 75% of incident cases present as non-muscle-invasive bladder cancer (NMIBC) with favorable survival but high recurrence rates, progression to muscle-invasive or mUC drastically worsens the prognosis; historically constrained to a 5% 5-year survival rate, contemporary mUC still demonstrates a limited median OS of 12 to 15 months, rarely exceeding 21 months in refractory settings [13,14].

Molecular studies have identified two main pathways of urothelial carcinogenesis that critically dictate the tumor’s immunological phenotype. Low-grade papillary tumors are often driven by activating mutations in the fibroblast growth factor receptor 3 (FGFR3) and HRAS genes. Notably, FGFR3 mutations—occurring in 15–20% of mUC and 40–50% of upper tract urothelial carcinomas (UTUC)—are strongly associated with a non-T-cell-inflamed (“cold”) tumor microenvironment, inherently limiting the efficacy of immunotherapy. In contrast, invasive forms exhibit abnormalities in suppressor genes like TP53 (present in ~70% of Muscle-Invasive Bladder Cancer [MIBC]) and RB1 (37%), alongside PIK3CA mutations (25%) and CDKN2A/B deletions (5–23%). While these TP53/RB1-altered tumors often correlate with a higher neoantigen load and a more inflamed (“hot”) phenotype, they simultaneously deploy potent compensatory immunosuppressive networks (e.g., Myeloid-Derived Suppressor Cells [MDSC] recruitment), ultimately facilitating immune evasion [15,16].

For decades, platinum-based chemotherapy—including gemcitabine-cisplatin (GC) or ddMVAC—served as the cornerstone of systemic treatment for mUC [17]. Recently, the therapeutic landscape shifted dramatically with the integration of ICIs, highlighted by the CheckMate 901 trial (concurrent GC with nivolumab) and the establishment of avelumab switch-maintenance therapy following platinum-based induction [18,19]. For cisplatin-ineligible patients, alternative frontline regimens include carboplatin or single-agent ICIs (e.g., pembrolizumab). However, despite these sequential chemo-immunotherapy approaches, standard cytotoxics frequently fail to induce durable immunogenic memory. Consequently, tumors undergo profound adaptive resistance, leading to disease progression and rendering mUC largely incurable at the metastatic stage. This inevitable resistance to standard cytotoxic and PD-1/PD-L1 pathways underscores an urgent clinical necessity for novel modalities—most notably ADCs—that can bridge potent, targeted cytotoxicity with the reactivation of an exhausted immune system [20].

The identification of predictive biomarkers is essential for the personalization of treatment in mUC. Although PD-L1 expression is commonly assessed, its value as a predictor of response to immunotherapy remains inconsistent in clinical trials. Other relevant indicators include high TMB, which may correlate with a better response to ICIs, and mutations in DNA repair genes (e.g., ERCC2). Currently, genetic alterations in FGFR2/3 are the only fully validated biomarker for eligibility for targeted therapy with erdafitinib. For ADC therapies such as EV, studies indicate widespread expression of Nectin-4 in UC, which currently eliminates the need for routine testing of this antigen level prior to treatment initiation [21].

EV represents a breakthrough in the treatment of patients with mUC after failure of standard lines of treatment. This drug is a conjugate directed against Nectin-4 (PVRL4). Nectin-4 is an adhesion protein that is highly overexpressed in UC cells, with minimal presence in healthy tissues, making it an ideal molecular target. EV delivers a cytotoxic payload, MMAE, directly to cancer cells. After internalization of the ADC complex by endocytosis, MMAE is released inside the cell, where it disrupts microtubule polymerization, leading to cell cycle arrest and apoptosis. An additional mechanism observed in experimental models is the bystander effect, whereby MMAE diffuses into neighboring cancer cells. While these immunomodulatory pathways present a compelling therapeutic rationale, clinical trials primarily demonstrate survival and response benefits, leaving the precise confirmation of these immune mechanisms in human subjects as an area for ongoing investigation [6].

## 4. Enfortumab Vedotin

EV belongs to the class of ADCs. ADCs represent a major advance in oncology, combining the specificity of monoclonal antibodies with highly potent cytotoxic agents. They offer a novel therapeutic approach with significant promise across multiple malignancies, including mUC [22,23]. EV is an ADC composed of a fully human IgG1-κ monoclonal antibody directed against Nectin-4, a cell-surface adhesion molecule commonly expressed in UC cells, conjugated to the microtubule-inhibiting MMAE. The antibody and cytotoxic agent are connected by a protease-cleavable maleimidocaproyl-valine-citrulline linker, enabling selective delivery of the cytotoxic drug to tumor cells [23,24].

**Figure 3:**
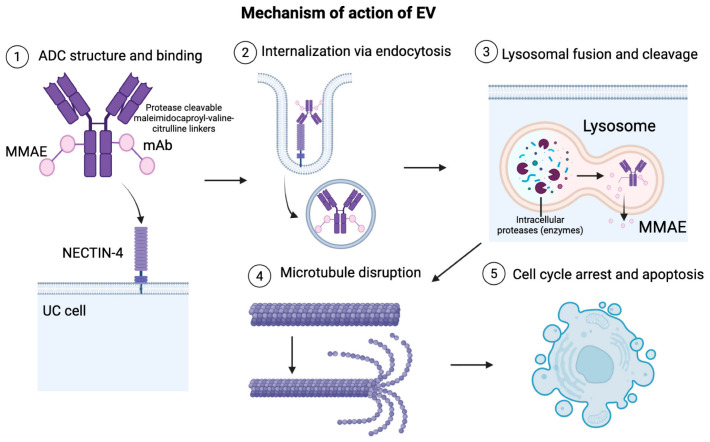
Mechanism of action of enfortumab vedotin (EV). (1) EV binds to Nectin-4 on.the urothelial carcinoma cell surface. (2) The complex is internalized via endocytosis. (3) Lysosomal proteases cleave the linker, releasing the cytotoxic payload (MMAE) into the cytoplasm. (4) MMAE disrupts the microtubule network, inhibiting polymerization. (5) This induces G2/M cell cycle arrest, leading to apoptosis and immunogenic cell death (ICD) [24,25,28].

ADCs are constructed to ensure preferential delivery of their cytotoxic payload to cancer cells, limiting collateral damage and increasing therapeutic potency [25]. UC cells show upregulated expression of Nectin-4, a protein that mediates cell–cell adhesion. While traditionally recognized primarily for this structural role, emerging evidence indicates that Nectin-4 (PVRL4) actively functions as an immune checkpoint within TME [26]. Recent studies demonstrate that Nectin-4 interacts with inhibitory receptors, most notably TIGIT, on the surface of T lymphocytes and Natural Killer (NK) cells, thereby suggesting a potential pathway for immune evasion in preclinical settings [27]. Consequently, the physical binding of EV to Nectin-4 may provide an additional layer of immune modulation; the targeted blockade of the hypothesized Nectin-4/TIGIT axis by the ADC could contribute to disrupting these mechanisms. Once bound to Nectin-4, EV is internalized into tumor cells, where MMAE is released by intracellular proteases. This disrupts the microtubule network, promoting cell cycle arrest and subsequent apoptosis. Crucially from an immunological perspective, MMAE-induced apoptosis is theorized to not be immunologically silent, as laboratory evidence demonstrates its capacity to trigger ICD and promote the release of damage-associated molecular patterns (DAMPs) into the tumor microenvironment, providing a potential rationale for driving adaptive immunity [28]. The mechanism of action of EV is shown in Figure 3.

The clinical development of EV in advanced UC has been driven by a sequence of pivotal clinical trials that progressively established its efficacy and safety across different treatment settings. Early phase clinical evaluation, including the first-in-human EV-101 trial, established the safety, tolerability, and initial evidence of antitumor activity of EV, thereby providing the foundation for subsequent clinical development [29]. This was followed by the multicenter, single-arm, phase II EV-201 trial, in which EV was administered as monotherapy at a dose of 1.25 mg/kg intravenously on days 1, 8, and 15 of each 28-day cycle to 89 patients. The study demonstrated clinically meaningful antitumor activity, with a confirmed ORR of 52% (46/89; 95% CI, 41–62), including CR in 20% and PR in 31% of patients. TRAEs occurred in all patients, with 16% leading to treatment discontinuation and 3% resulting in death. One additional treatment-related death due to pneumonitis occurring more than 30 days after the last EV dose was not included in this percentage [30]. These results supported the initial approval of EV by the Food and Drug Administration (FDA) in 2019 for patients with locally advanced or mUC previously treated with platinum-based chemotherapy and PD-1/PD-L1 inhibitors [31]. In July 2021, the FDA further expanded this indication to include patients who had previously received a PD-1 or PD-L1 inhibitor and platinum-containing chemotherapy, or those who were ineligible for cisplatin-based chemotherapy and had received prior therapy [32].

Subsequent investigation in the EV-103/KEYNOTE-869 trial evaluated EV in combination with pembrolizumab in cisplatin-ineligible patients, demonstrating a high ORR of 68%, including CR in 12% of patients, with a median DoR of 22 months [33]. While this profound clinical activity provided encouraging preliminary evidence of potential immune synergy, the non-randomized design of these early cohorts necessitated definitive validation in phase III settings, ultimately contributing to its accelerated FDA approval in 2023. Further evidence from phase III trials reinforced this paradigm. While the EV-301 study established EV monotherapy as a standard option by demonstrating a significant OS improvement over chemotherapy in previously treated patients, the phase III EV-302/KEYNOTE-A39 trial marked a fundamental shift in mUC management. By demonstrating superior clinical outcomes for the EV and pembrolizumab combination compared to platinum-based chemotherapy in previously untreated patients, the trial validated the hypothesis that EV-mediated ICD successfully primes the immunosuppressive TME for PD-1 blockade. These results led to full FDA approval of the combination regimen in 2023, establishing a new first-line standard of care driven by targeted chemo-immunotherapy synergy [34].

## 5. Results

### 5.1. EV-103

#### 5.1.1. Phase IB/II

The EV-103 phase IB/II study involved 149 patients who were ineligible for chemotherapy and were divided into two groups. Group I received EV + pembrolizumab (n = 76) and group II received EV monotherapy (n = 73).

In group I (EV + pembrolizumab), ORR was observed in 64.5%, with CR in 10.5%, PR in 53.9%, and PD in 7.9% of patients. In group II (EV monotherapy), the corresponding figures were 45.2%, 4.1%, 41.1%, and 9.6%. Given that the EV-103 trial evaluated these cohorts without direct head-to-head randomization, these findings should be interpreted descriptively as hypothesis-generating data rather than definitive proof of biological synergy or clinical superiority.

During the assessment of tolerance and safety, TRAEs of grade 3 and higher were observed in 63.2% of patients in group I and in 47.9% in group II. The most common any-grade TRAEs in patients treated with EV + pembrolizumab were fatigue (56.6%), peripheral sensory neuropathy (51.3%), alopecia (46.1%), maculopapular rash (46.1%), pruritus (39.5%), and dysgeusia and weight decreased (30.3%). In contrast, in the group receiving EV alone, the most common Grade 3 or higher adverse events were peripheral sensory neuropathy (43.8%), fatigue (39.7%), decreased appetite (38.4%), alopecia (35.6%), dysgeusia (34.2%), and nausea (34.2%). The study reported deaths related to adverse effects of therapy: 3 in group I (3.9%) and 2 in group II (2.7%). While the combination therapy inherently presented a higher toxicity profile due to additive toxicities and dual-drug administration, both EV monotherapy and the combination regimen remained generally well tolerated by patients [35]. Table 2 summarizes efficacy outcomes.

#### 5.1.2. PROs

In cohort K of the EV-103 study, patients receiving EV as monotherapy (EV, n = 61) or in combination with pembrolizumab (EV + Pembrolizumab; n = 65) were analyzed for PRO. Quality of life and symptom severity were analyzed using the EORTC QLQ-C30, EORTC QLQ-BLM30, and Brief Pain Inventory—Short Form (BPI-SF) questionnaires.

Overall quality of life remained stable in both treatment groups during therapy. In the EV + pembrolizumab arm, slight changes from baseline were observed at weeks 8, 12, and 24 (−0.82; −1.88; 1.59), while in the EV monotherapy arm, these values were 2.70; −0.62 and 2.03, respectively. Both groups showed improvement in emotional functioning and pain reduction during treatment. In the EV + pembrolizumab group, clinically significant improvement in pain was observed at weeks 12 and 24 (−14.41 and −14.99), while in the EV group it was observed at weeks 8, 12, and 24 (−10.11; −10.55; −12.55). An early, transient worsening of some symptoms, such as fatigue, loss of appetite, and diarrhea, was observed around week 3 of therapy, but these values later returned to baseline levels.

The BPI-SF analysis showed improved pain control, particularly in the EV + pembrolizumab group, where a clinically significant reduction in worst pain was observed at week 24 (−2.07). A sustained improvement in pain was reported by 76.7% of patients in the combination therapy arm and 65.4% in the EV monotherapy arm, with a median time to improvement of 1.2 and 1.0 months, respectively. Improvement in worst pain on the BPI-SF scale was observed in 73.9% of patients in the EV + pembrolizumab group and in 47.7% of patients in the EV group [36]. Table 3 shows the main PROs from cohort K.

### 5.2. EV-301

#### 5.2.1. Phase III

The EV-301 phase III randomized clinical trial involved 608 patients with locally advanced or metastatic UC who had previously received platinum-based chemotherapy and whose disease had progressed during or after treatment with a PD-1/PD-L1 inhibitor. The patients were randomly divided into two groups: the study group (n = 301) receiving EV at a dose of 1.25 mg/kg on days 1, 8, and 15 of a 28-day cycle, and the control group (n = 307) receiving standard chemotherapy in 21-day cycles based on docetaxel (75 mg/m^2^ i.v.), paclitaxel (175 mg/m^2^ i.v.) or vinflunine (320 mg/m^2^ i.v.). The analysis showed a 30% reduction in the risk of death in the study group compared to the control group (Hazard Ratio (HR) = 0.70 [95% CI: 0.56–0.89]; *p* = 0.00142). In addition, it improved progression-free survival by reducing the risk of progression or death by 38% (HR = 0.62 [95% CI: 0.51–0.75]; *p* < 0.00001. The most important key results indicating the superiority of EV over classic therapeutic treatments are presented in Table 4. It is also worth noting the benefit in terms of overall survival in the clinical subgroup with liver metastases, which includes patients with a particularly poor prognosis, in which high efficacy was demonstrated (HR-0.68). Crucially, a significant reduction in the risk of death was observed regardless of the patient’s prior response to PD-1/PD-L1 inhibitors (HR = 0.66 for the non-responsive group). From an immunological perspective, this efficacy in ICI-refractory tumors is highly significant; it demonstrates that EV can effectively target and eradicate tumor clones that have developed adaptive immune resistance and successfully evaded T-cell-mediated clearance. These results confirm that EV therapy serves not merely as a cytotoxic rescue option, but as a critical intervention capable of overcoming the severe limitations of an exhausted immune microenvironment.

The safety of both therapies was comparable. Adverse events in the EV group occurred in 93.9%, including grade 3 and higher in 51.4%, and in the chemotherapy group, 91.8% and 49.8%, respectively. The most common adverse events in the study group were alopecia, peripheral sensory neuropathy, pruritus, fatigue, and decreased appetite. In the control group, the most common side effects were alopecia, decreased appetite, fatigue, and anemia. In addition, patients receiving EV experienced a reduction in hematological side effects such as neutropenia (6.4% vs. 34.7%) and anemia (3.1% vs. 14.3%) [37].

#### 5.2.2. 24-Month Follow-Up

The 24-month assessment of the EV-301 study confirmed long-term results consistent with those of the primary analysis. It was shown that mOS and mPFS were longer with EV compared to chemotherapy. They were 12.91 months and 8.94 months, and 5.55 months and 3.71 months, respectively. In addition, the risk of death was reduced by 30% (HR = 0.70, *p* = 0.00015), and the risk of disease progression or death was reduced by 37% in the EV group (HR = 0.63, *p* < 0.00001). Furthermore, approximately 30% of patients receiving EV survived for 2 years, compared to only 20% in the chemotherapy group. The overall response rate in the study group was 41.32%, compared to only 18.58% in the control group (*p* < 0.0001). DC was observed in 71.88% of patients receiving EV and 53.38% receiving chemotherapy (*p* < 0.001).

The incidence of TRAEs was also similar after 24 months of follow-up compared to the primary analysis. Grade 3 TRAEs were observed in the EV group in the interim analysis in 51.4% of patients and in the long-term analysis in 52.4%. Similarly, in the chemotherapy group, this was 49.8% and 50.5% [7].

#### 5.2.3. Japanese Subgroup

In a study evaluating the Japanese subgroup of the EV-301 study, 86 patients were evaluated, 36 of whom received EV and 50 received chemotherapy. The results showed that in the study group, OS was 15.18 months (HR = 0.437), PFS was 6.47 months (HR = 0.464), and ORR was 34.4%. The corresponding results in the control group were 10.55 months, 5.39 months, and 21.3%. In addition, the analyzed subgroup showed a lower incidence of TRAEs, 63.9% in patients receiving EV vs. 75% in those receiving chemotherapy [38].

### 5.3. EV-302 KEYNOTE-A39

#### 5.3.1. Phase III

A total of 886 patients with untreated laUC participated in phase 3 RCTs. The patients were divided into two groups: the study group receiving EV together with pembrolizumab (n = 442) and the control group receiving chemotherapy based on gemcitabine and cisplatin or carboplatin (n = 444). In the study arm, EV was administered intravenously at a dose of 1.25 mg/kg body weight on days 1 and 8 of each 21-day cycle, while pembrolizumab was administered at a dose of 200 mg intravenously on day 1 of the cycle. In the control arm, patients received gemcitabine as an intravenous infusion at a dose of 1000 mg/m^2^ on days 1 and 8 and cisplatin as an intravenous infusion at a dose of 70 mg/m^2^ or carboplatin as an intravenous infusion at a dose corresponding to an Area Under the Curve (AUC) of 4.5–5 mg·ml^−1^·min, calculated according to the Calvert formula, administered on day 1 of each 3-week cycle. The results showed that EV therapy was more beneficial and had fewer adverse events. The risk of disease progression or death was 55% lower in the EV group compared to chemotherapy (HR = 0.45. *p* < 0.001). A 53% reduction in the risk of death was observed (HR = 0.47, *p* < 0.001). In addition, the median PFS was 12.5 months in the study group, compared with 6.3 months in the control group. The median OS was 31.5 months and 16.1 months, respectively. Additionally, ORR was 67.7% versus 44.4%, and CR reached an unprecedented 29.1% versus 12.5%. This striking near-tripling of the complete response rate compared to standard cytotoxic regimens provides compelling clinical evidence that EV-induced immunogenic cell death synergizes profoundly with PD-1 blockade, effectively transforming the anti-tumor immune response. Regarding safety, the combination therapy demonstrated a favorable profile; grade 3 and higher adverse events occurred in 55.9% of patients in the EV + P study group, compared to 69.5% in the chemotherapy control group. Table 5 shows the percentage occurrence of individual grade 3 and higher adverse events, highlighting a significant reduction in the incidence of severe hematological toxicities with the EV + P regimen [39].

#### 5.3.2. Subgroup Analyses

Analysis of the EV-302 study confirmed the benefits of EV + P in various patient subgroups, consistent with earlier results for the Intention-to-Treat (ITT) population. In the overall population, EV + P demonstrated a significant and clinically meaningful improvement in PFS and OS compared to chemotherapy in patients with previously untreated la/mUC, with a 55% reduction in the risk of disease progression or death and a 53% reduction in the risk of death.

In subgroup analyses, EV + P improved ORR regardless of eligibility for cisplatin treatment. In cisplatin-eligible patients, ORR was 70.8% with EV + P vs. 53.0% with chemotherapy, and CR was 32.5% vs. 15.5%. In the cisplatin-ineligible group, the ORR was 63.9% vs. 34.9%, and the CR was 24.7% vs. 9.1%. The risk of disease progression or death was reduced by 52% in cisplatin-eligible patients and by 57% in cisplatin-ineligible patients, while the risk of death was reduced by 47% and 57%, respectively.

The EV + P safety profile was consistent across all subgroups and the ITT population, with no new safety signals. TRAEs of any grade occurred in 94–98% of patients, and TRAEs leading to discontinuation of treatment affected 26–45% of patients treated with EV and 19–30% of patients treated with pembrolizumab.

EV + P outperformed chemotherapy across all metastatic subgroups, including cisplatin-eligible/ineligible patients and those with liver, visceral, or lymph node-limited metastases. The highest ORR was observed in lymph node-confined disease, and the longest DOR in visceral and no liver metastasis subgroups, confirming the sustained efficacy of EV + P. These results are presented in Table 6 [8].

#### 5.3.3. 2.5 Year Follow-Up

After a median follow-up of approximately 2.5 years in the EV-302 study, first-line EV + P treatment continued to show higher efficacy than chemotherapy, nearly doubling the median PFS and more than doubling the median OS. The PFS and OS benefits were consistent across all subgroups, including patients eligible and ineligible for cisplatin treatment. Responses to therapy were durable, with a median DOR of approximately two years, and among patients with a complete clinical response (cCR), 74.3% maintained cCR at 24 months. The safety profile remained stable with no new signals, and the incidence and severity of TRAEs, including neuropathy and ocular disorders, were consistent with the previous analysis. These results confirm the highly durable benefit of EV + P over chemotherapy. Crucially, the ability to maintain complete clinical responses over such an extended period strongly suggests the successful establishment of long-term, adaptive anti-tumor immune memory, firmly reinforcing the use of this synergistic regimen as the standard first-line treatment in patients with laUC or mUC [40].

#### 5.3.4. PROs

It is important to acknowledge that the current clinical evidence base for EV in advanced UC is predominantly derived from a limited number of pivotal clinical trial programs, specifically EV-103, EV-301, and EV-302. While these trials provide robust and high-quality data that have reshaped the treatment paradigm, the reliance on a small number of core studies may limit the generalizability of these findings to broader, more diverse real-world patient populations who often present with multiple comorbidities and lower performance status.

Analysis of PROs from the EV-302 phase III trial showed a beneficial effect of EV therapy in combination with pembrolizumab on quality of life and pain intensity compared to platinum-based chemotherapy. The study included 731 patients who completed at least one PRO questionnaire (376 in the EV + pembrolizumab group, 355 in the chemotherapy group). The median time to pain progression was 14.2 months in the combination therapy group and 10.0 months in the chemotherapy group (HR = 0.92; 95% CI: 0.72–1.17), which was not statistically significant. In contrast, the change in the intensity of the most severe pain in the BPI-SF questionnaire up to week 26 was significantly more favorable in the EV + pembrolizumab group (LS mean −0.74 vs. −0.36; difference −0.38; 95% CI: −0.64 to −0.12; *p* = 0.0037).

Quality of life assessment according to EORTC QLQ-C30 GHS/QoL showed the superiority of combination therapy, with a mean change at week 26 of +2.54 points compared to chemotherapy (95% CI: 0.41–4.67). In patients with moderate or severe pain at baseline, clinically meaningful improvements were observed in both pain intensity (LS mean −0.53; 95% CI: −1.03 to −0.02; *p* = 0.041) and quality of life (LS mean +4.77; 95% CI: 1.24–8.29; *p* = 0.0083). These results suggest that EV treatment in combination with pembrolizumab not only does not worsen the quality of life of patients, but in some patients may lead to an improvement in pain symptoms and overall health compared to chemotherapy [41].

### 5.4. Summary of Clinical Trials

Table 7 summarizes the key studies included in this review, confirming the clinical benefits of EV in UC therapy.

## 6. Discussion

Both laUC and mUC continue to pose a significant clinical challenge due to the aggressive course of the disease and limited treatment options in some patient populations. For many years, platinum-based chemotherapy has been the mainstay of systemic treatment. However, it should be noted that approximately half of patients with laUC or mUC are not eligible for cisplatin treatment due to their general condition, comorbidities, or renal impairment. In this group, the standard therapeutic option remains gemcitabine with carboplatin, which is characterized by a relatively low ORR of approximately 36–42%, a short DoR (6.3–7.1 months), and limited treatment tolerance. Therefore, there is a significant need to develop new, more effective therapeutic strategies, particularly for patients ineligible for cisplatin [35].

In recent years, significant progress in the treatment of UC has been made with the introduction of ADCs, which enable selective delivery of a cytotoxic payload directly to cancer cells. EV, which targets Nectin-4, a protein highly expressed in UC cells, is one of the most important representatives of this class of drugs. Beyond direct cytotoxicity, EV-induced apoptosis has been shown to elicit ICD in preclinical models. This mechanism is crucial, as the release of tumor-associated antigens and DAMPs following MMAE-mediated cell disruption enhances the recruitment and activation of tumor-infiltrating lymphocytes. By favorably altering the immunosuppressive tumor microenvironment, EV likely serves as an in situ “primer” that facilitates a more robust response to immune checkpoint inhibitors. This provides a strong biological rationale for the clinical synergy observed in the EV-302 trial, suggesting that the benefits of EV + pembrolizumab extend beyond simple additive effects. However, overcoming therapeutic resistance within this milieu remains a complex challenge, particularly in tumors characterized by FGFR alterations. Recent evidence suggests that FGFR-mediated pathways can actively suppress anti-tumor immunity, thereby limiting the efficacy of ICIs and standard chemotherapy. While targeting these alterations with novel selective pan-FGFR inhibitors yields encouraging objective response rates in biomarker-selected patient populations, these agents also introduce substantial safety concerns, such as frequent treatment interruptions due to adverse events. Therefore, carefully balancing the distinct efficacy and safety profiles of novel targeted agents alongside emerging combination strategies is essential to optimize outcomes [4,42].

Looking ahead, these challenges underscore the necessity for future clinical research to prioritize several key areas. Ongoing phase III trials are essential to define the optimal role of EV in earlier-stage disease, particularly in the perioperative and neoadjuvant settings. Furthermore, there is a critical need to refine biomarker-driven patient selection to identify those most likely to benefit from EV-based regimens. Investigating the molecular mechanisms of acquired resistance will be equally vital for developing next-generation therapeutic strategies, and the exploration of novel combination strategies, extending beyond current immune checkpoint inhibitors, remains a high priority to further improve outcomes in advanced urothelial carcinoma.

The clinical development of EV reflects a stepwise evolution across treatment lines and therapeutic strategies. Early-phase studies (EV-201) first demonstrated clinically meaningful antitumor activity in heavily pretreated patients, which was subsequently validated in the phase III EV-301 trial, establishing EV as a standard option in later-line settings. While these trials firmly established EV as a standard in later-line settings, its role must be interpreted in the context of other established standards, such as maintenance avelumab for patients with stable disease following platinum-based chemotherapy. Unlike sequential approaches, the integration of EV-based combinations in the first-line setting, as evidenced by the EV-302 trial, represents a paradigm shift that may challenge the necessity of traditional maintenance strategies. Subsequent expansion into earlier treatment settings was achieved through combination strategies, particularly in EV-103, which provided a preliminary, hypothesis-generating rationale for integrating EV with immune checkpoint inhibitors. The pivotal EV-302/KEYNOTE-A39 trial redefined first-line treatment standards, demonstrating superior OS and PFS compared to platinum-based chemotherapy and marking a paradigm shift in the management of laUC or mUC. These phase III results demonstrate the robust clinical efficacy of this approach, and while they are consistent with the hypothesis that EV-mediated cytotoxicity may prime the tumor microenvironment for immune checkpoint inhibition, the primary data confirm clinical survival benefits rather than direct immune remodeling. These results have established this combination as the new first-line standard of care for patients with laUC or mUC.

The introduction of the EV regimen in combination with pembrolizumab (EV + P) represents a fundamental paradigm shift in the treatment of laUC, which for three decades has relied solely on chemotherapy. Importantly, this shift extends beyond efficacy alone and reflects improved tolerability and broader applicability in real-world patient populations. This is particularly important for patients who are not eligible for cisplatin, for whom previous options, such as carboplatin with gemcitabine, offered limited response duration and poorer prognosis. These data suggest that EV + P is becoming the new preferred standard of care, regardless of a patient’s eligibility for chemotherapy.

In everyday clinical practice, UC mainly affects older people with multiple comorbidities, in whom aggressive chemotherapy often leads to severe hematological complications. The use of EV allows for a significant reduction in the risk of neutropenia and anemia compared to classic cytostatics. This makes this therapy a safer alternative, avoiding frequent hospitalizations and loss of organ reserve in seniors.

Emerging real-world evidence (RWE) further reinforces the clinical effectiveness and safety profile of EV, demonstrating outcomes that are largely consistent with those observed in controlled clinical trials across broader and more heterogeneous patient populations, including elderly individuals and those with significant comorbidities. Importantly, these real-world data provide complementary insights into treatment feasibility and tolerability outside the stringent inclusion criteria of clinical trials, thereby bridging the translational gap between experimental settings and routine clinical practice [43,44,45].

RWE also encompasses detailed case reports that highlight the clinical benefit of EV in diverse patient populations. For instance, a recently published case describes a patient with advanced UC who achieved a durable response to EV following prior platinum-based chemotherapy and immunotherapy. This observation not only underscores the antitumor efficacy of EV in heavily pretreated patients but also emphasizes its manageable safety profile in real-world settings. Incorporating such individual patient experiences adds a practical and translational dimension to the discussion, illustrating how EV can achieve meaningful clinical outcomes while maintaining tolerability, particularly in populations often underrepresented in clinical trials [46].

Importantly, EV should be considered within the broader context of emerging ADCs. Disitamab vedotin (RC48-ADC), targeting Human Epidermal Growth Factor Receptor 2 (HER2), has demonstrated encouraging efficacy in UC, with real-world studies reporting high objective response rates (ORR ~69%) and a median PFS of ~24 months in treated populations; OS data remain immature but 12- and 24-month survival rates are favorable, and ongoing studies continue to mature [47].

At the same time, EV-based regimens must be critically evaluated against other established therapeutic strategies, including platinum-based chemotherapy followed by avelumab maintenance, as well as platinum-based chemotherapy combined with nivolumab. Although EV + P demonstrates substantial efficacy, with high response rates and improved tolerability in real-world populations, direct comparative trials are lacking. Consequently, evaluating the relative efficacy and safety of these frontline regimens requires rigorous statistical modeling rather than informal cross-trial assessments. Treatment selection should therefore be individualized, integrating patient comorbidities, prior therapies, molecular biomarkers, and patient preferences.

Regarding the optimal sequencing of EV, it remains one of the most pressing challenges in the management of mUC. Currently, there is a lack of prospective, head-to-head clinical data to guide the order of administration between EV-based regimens, platinum-based chemotherapy, and subsequent therapies such as immune checkpoint inhibitors or alternative ADCs. While the integration of EV + pembrolizumab has moved to the frontline setting, the appropriate sequencing for patients who progress on this regimen—or for those who receive platinum-based therapy followed by avelumab maintenance—is yet to be established. Ongoing research is urgently needed to determine whether prior exposure to ADCs or ICIs influences the efficacy of subsequent agents. In clinical practice, sequencing decisions must currently be personalized, balancing the depth of response, safety profile, and the availability of salvage therapies, until standardized algorithms can be defined by future clinical trials.

Treatment selection in laUC or mUC is increasingly complex, as multiple first-line strategies are now available. Standard platinum-based chemotherapy has historically been the backbone of therapy, with a median OS of approximately 15 months and median PFS around 7–8 months in advanced disease populations [48]. Recent studies incorporating immunotherapy have reshaped this landscape. The CheckMate 901 trial demonstrated that adding nivolumab to gemcitabine and cisplatin improved median OS to 21.7 months versus 18.9 months with chemotherapy alone (HR 0.78), while also increasing objective response rate (57.6% vs. 43.1%) and complete response rates (21.7% vs. 11.8%) [49]. In a complementary approach, the JAVELIN Bladder 100 trial established the role of avelumab maintenance after completion of platinum-based chemotherapy, showing a median OS of approximately 23.8 months versus 14.3 months with best supportive care alone [50].

Although this article focuses on EV, it is important to contextualize its use within the broader treatment landscape of UC. Platinum-based chemotherapy followed by avelumab maintenance, as well as platinum plus nivolumab, remain key standard-of-care options for eligible patients. EV, particularly in combination with pembrolizumab, offers a compelling alternative with high efficacy and favorable tolerability, especially for patients who are cisplatin-ineligible or have comorbidities, highlighting the need for individualized therapy selection based on patient and disease characteristics [51,52].

However, direct cross-trial comparisons between these milestones are methodologically limited due to substantial variations in patient settings—specifically, comparing primary frontline combinations with switch-maintenance strategies. To address this heterogeneity, a recent network meta-analysis comprehensively evaluated these emerging first-line regimens. These findings provide a robust statistical framework, confirming that while immune checkpoint inhibitor plus antibody–drug conjugate (ICI + ADC) combinations yield the most substantial improvements in overall survival and progression-free survival across both cisplatin-eligible and cisplatin-ineligible populations (OS: HR 0.47; PFS: HR 0.45), they also carry an increased risk of grade ≥ 3 adverse events compared to less intensive treatment pathways [53].

In this context, treatment selection is becoming increasingly complex and should incorporate not only clinical factors such as performance status and comorbidities but also emerging molecular and biomarker-driven criteria. The identification of patients most likely to benefit from EV-based therapy versus alternative strategies remains a critical unmet need.

When analyzing the safety profile, it is important to note the significant differences in the type of toxicity between new therapies and classic chemotherapy. While EV regimens demonstrate significantly lower rates of severe hematological complications than platinum-based chemotherapy, they require vigilant monitoring for distinct payload- and target-driven toxicities. Specifically, key adverse events include peripheral sensory neuropathy (occurring in ~50% of patients across trials; Grade ≥3 in 3–5%), dermatologic toxicities such as maculopapular rash (~45%; Grade ≥3 in ~8%), MMAE-induced hyperglycemia (Grade ≥3 in 5–10%), and ocular disorders including dry eye and corneal irritation. Crucially, these toxicities directly impact treatment delivery; in the EV-302 trial, adverse events led to EV discontinuation in approximately 35% of patients and dose reductions in over 50%, underscoring the necessity of proactive toxicity management.

Furthermore, these survival benefits are supported by favorable PROs. In the pivotal EV-302 trial, using EORTC QLQ-C30 and QLQ-EN24 instruments, patients receiving EV + pembrolizumab reported a significant delay in time to pain progression compared to those treated with chemotherapy. Crucially, global health status and quality-of-life scores remained stable over time in the combination arm, whereas patients receiving platinum-based chemotherapy experienced a transient deterioration during active treatment. This confirms that the combination regimen not only extends survival but effectively controls disease-related symptoms and preserves patient quality of life.

HER2 and Nectin-4 expression therefore represent complementary biomarkers that can inform ADC selection: high HER2 expression may favor RC48, while Nectin-4 expression directs the use of EV. In practice, the choice between EV and RC48 should integrate tumor biology, prior therapies, comorbidities, and patient treatment goals, as well as considerations of toxicity profiles and patient preference. Optimal sequencing of ADCs remains an important consideration, particularly for patients eligible for multiple ADC-based options. Tailoring therapy based on tumor biology and patient characteristics is likely to maximize efficacy, minimize toxicity, and inform future combination strategies in UC [54].

Beyond the metastatic setting, ongoing clinical trials are actively investigating the role of EV in earlier stages of disease, including neoadjuvant and perioperative approaches (e.g., EV-203, EV-303). Recently reported results from EV-304 demonstrate promising efficacy and a favorable safety profile in the perioperative setting supporting the potential of EV to expand its therapeutic scope and shift treatment paradigms toward earlier interventions.

In the context of further refinement of EV therapy, increasing attention is being paid to predictive response biomarkers and resistance mechanisms. Nectin-4 expression is a key determinant of EV sensitivity, with data indicating that patients with high Nectin-4 expression show better therapeutic responses. In addition, the composition of the TME, including the presence of immunosuppressive cells, may affect the efficacy of ADCs by limiting drug internalization or cytotoxic activity. Laboratory studies and clinical sample analyses also indicate the development of resistance to ADCs through mechanisms such as reduced Nectin-4 expression, increased expression of efflux transporters for MMAE, and adaptive changes in cell signaling associated with tumor cell survival. Understanding these mechanisms not only allows for better patient selection for therapy, but also paves the way for new combination strategies, such as combining EVs with immune checkpoint inhibitors or transporter inhibitors, which could potentially overcome resistance and improve treatment outcomes [55,56].

Furthermore, resistance to EV appears to be a multifactorial process involving alterations in antigen expression, intracellular drug trafficking, and microtubule dynamics. The tumor microenvironment may additionally modulate ADC efficacy through impaired drug delivery and immune-mediated mechanisms, highlighting the need for integrated biomarker-driven approaches. Specifically, the accumulation of immunosuppressive cell subsets within the TME, such as myeloid-derived suppressor cells (MDSCs) and regulatory T-cells (Tregs), may hinder the immune-stimulating effects of ADC-induced ICD, thereby limiting the long-term efficacy of combination strategies like EV plus pembrolizumab.

An additional unresolved question concerns the optimal sequencing of therapies, particularly in patients exposed to EV in earlier lines of treatment. The impact of prior ADC exposure on subsequent treatment efficacy remains poorly defined and warrants further investigation. Addressing these challenges is critical to fully realizing the clinical potential of EV and related ADC therapies, and for advancing personalized treatment strategies in UC.

In summary, available clinical data indicate that EV significantly changes the therapeutic landscape of UC treatment. The integration of ADC-based therapies with immunotherapy represents a transformative shift in UC management, redefining treatment paradigms across multiple disease stages. At the same time, further translational and clinical research remains essential to optimize therapeutic strategies, identify biomarkers of treatment response, and determine the optimal sequence of therapies. However, key challenges remain, including optimal patient selection, biomarker development, and therapeutic sequencing in an increasingly complex treatment landscape. Addressing these challenges will be critical to fully realizing the clinical potential of EV and related therapies.

Taken together, these findings position EV as a cornerstone of contemporary UC therapy and a key driver of the transition toward more personalized and mechanism-based treatment strategies. EV is now central to contemporary UC therapy, improving outcomes, tolerability, and enabling biomarker-driven personalization of treatment.

## 7. Limitations

In this systematic review, several factors should be taken into account when interpreting the results obtained. First, the number of available randomized clinical trials evaluating the efficacy of EV in the treatment of UC is still relatively limited, which is due to the relatively recent introduction of this therapy into clinical practice. In addition, the patient populations included in the analyzed studies were largely selected and mainly included patients in good general health and with a limited number of comorbidities. Therefore, these populations may not fully reflect the actual diversity of patients encountered in everyday clinical practice, especially older people with multiple comorbidities or those who have previously undergone intensive treatment.

Secondly, the observation period in some studies was relatively short, which makes it difficult to fully assess the long-term efficacy and safety of the therapy. In some studies, selected endpoints, such as patients’ quality of life or long-term treatment tolerance, were not analyzed in a uniform manner. Furthermore, the lack of standardized immune-monitoring protocols across these trials limits our ability to definitively characterize the long-term impact of EV on the restoration of immune surveillance and the durability of the anti-tumor memory response. In addition, this review included only publications in English, which may potentially involve a risk of selection bias.

Despite these limitations, an effort was made to present the current state of knowledge as objectively as possible, highlighting both the therapeutic potential of EV and areas requiring further research.

## 8. Conclusions

UC, especially in advanced and metastatic stages, remains a significant clinical challenge due to its aggressive course, high risk of progression, and limited therapeutic options in some patients. EV, as an ADC targeting Nectin-4, represents a significant advance in the development of targeted therapies for the treatment of this cancer. Available clinical data indicate a significant improvement in key treatment efficacy parameters, including overall survival and progression-free survival, compared to standard chemotherapy.

Particularly promising results have been obtained with the combination of EV and immune checkpoint inhibitors such as pembrolizumab, where the induction of immunogenic cell death by EV is hypothesized to complement and potentially enhance the anti-tumor immune response, suggesting a potential ability of EV to help overcome baseline immune evasion. However, these proposed immunomodulatory mechanisms, as well as the optimal integration of EV into evolving treatment algorithms, require further prospective validation. At the same time, the dynamic development of ADC-based therapies underscores the growing importance of precision medicine in oncology.

Despite these promising clinical trial results, further research into the complex mechanisms of action of EV, particularly its role in remodeling the immune microenvironment, remains essential to fully assess long-term efficacy and safety, and to optimize precision therapeutic strategies.

## Figures and Tables

**Table 1:** Eligibility criteria based on the PICO framework.

Component	Criteria
Population (P)	Adult patients diagnosed with UC, any gender
Intervention (I)	EV as monotherapy or in combination with immunotherapy (pembrolizumab)
Comparison (C)	Standard chemotherapy (platinum, docetaxel, paclitaxel) or other RCTs arms (EV vs. EV + pembrolizumab)
Outcomes (O)	Efficacy: ORR, CR, PR, SD, PD, PFS, OS, DoR; Safety: TRAEs
Study Design	Prospective clinical trials (phase I–III), including randomized trials, phase Ib/II studies, predefined subgroup analyses, long-term follow-up reports, and PRO analyses linked to parent trial programs
Publication years	2020–2026
Language	English
Exclusion criteria	Review publications, preclinical studies (animal or cellular models), abstracts or conference posters without complete data, studies not reporting efficacy or safety outcomes of EV treatment

**Table 2:** Exploratory results from the EV-103 study (Phase IB/II) in patients receiving EV monotherapy or EV in combination with pembrolizumab. EV-103 was a non-randomized, phase IB/II study. Data are presented for descriptive purposes only and should not be interpreted as direct comparative efficacy [35].

Parameter	EV + Pembrolizumab (n = 76)	EV (n = 73)
Responders	49	33
Progression events	13	14
ORR %	64.5%	45.2%
DOR > 12 months %	65.4%	56.3%
Median OS months	22.3	21.7
PFS events	31	38
OS events	20	26
SD %	22.4	34.2

**Table 3:** PROs results from cohort K of the EV-103 study [36]. Quality of Life (Qol).

Parameter	EV + Pembrolizumab	EV Monotherapy
QoL stability (QLQ-C30)	stable up to 24 weeks	stable up to 24 weeks
Change in QoL (8/12/24 weeks)	−0.82/−1.88/1.59	2.70/−0.62/2.03
Pain improvement (QLQ-C30)	−14.41 (12 weeks), −14.99 (24 weeks)	−10.11 (8 weeks), −10.55 (12 weeks), −12.55 (24 weeks)
Reduction in worst pain (BPI-SF)	−2.07 (24 weeks)	stable/slight improvement
Durable pain improvement	76.7%	65.4%
Median time to improvement	1.2 months	1.0 month

**Table 4:** Key results of the EV-301 study indicating the efficacy of EV [37]. IO-immuno-Oncology.

Clinical Parameter	EV	Chemotherapy	Statistical Significance
Median OS (months)	12.88	8.97	HR = 0.70 (*p* = 0.001)
Median PFS (months)	5.55	3.71	HR = 0.62 (*p* < 0.001)
12-month OS (%)	51.5%	39.2%	-
ORR (%)	40.6%	17.9%	*p* < 0.001
DCR (%)	71.8%	53.4%	-
CR (%)	4.9%	2.7%	-
OS in Liver Metastases	Favors EV	-	HR = 0.68
OS in IO Non-responders	Favors EV	-	HR = 0.66

**Table 5:** Grade 3 and higher TRAEs [39].

TRAEs	EV + Pembrolizumab	Chemotherapy
Anemia	3.4%	31.4%
Neutropenia	4.8%	30%
Thrombocytopenia	0.5%	19.4%
Diarrhea	3.6%	0.7%
Fatigue	3.0%	4.2%
Nausea	1.1%	2.8%
Peripheral sensory neuropathy	3.6%	0%
Pruritus	1.1%	0%
Maculopapular rash	7.7%	0%
Hyperglycemia	5%	0%
Decreased appetite	1.1%	1.4%

**Table 6:** Results in patients divided into subgroups based on the presence of metastases in patients with UC [8]. Chemotherapy (CH), not estimable due to insufficient events at data cutoff (NE).

Subgroup Parameter	Cisplatin– Eligible n = 244	Cisplatin– Ineligible n = 198	Liver Metastases n = 199	No Liver Metastases n = 687	Visceral Metastases n = 636	Lymph Nodes Only n = 207
PFS (EV + P)	12.5 months	10 months	8.2 months	16.4 months	10.4 months	NE
PFS (CH)	6.3 months	6.2 months	6 months	6.4 months	6.2 months	8 months
OS (EV + P)	NE	NE	19.1 months	NE	25.6 months	NE
OS (CH)	15.9 months	13.6 months	10.1 months	17.9 months	13.6 months	27.5 months
ORR (EV + P)	70.8%	63.9%	60%	70%	64.1%	77.5%
ORR (CH)	53%	34.9%	41.4%	45.3%	39.6%	53.4%
DOR (EV + P)	NE	NE	12.9 months	NE	20.2 months	NE
DOR (CH)	8.3 months	6.6 months	5 months	8.3 months	6.1 months	12.5 months

**Table 7:** Summary table of clinical trials evaluating the efficacy and safety of EV in UC therapy.

Year	Type of Examination	Intervention	Population	Results	Refs.
2023	Phase IB/II Non-randomized	EV monotherapy/EV + pembrolizumab	149	ORR 45.2% vs. 64.5%; CR 4.1% vs. 10.5%; median DoR 7.3 vs. 22.1 months	[35]
2024	Phase IB/II Non-randomized PROs	EV monotherapy/EV + pembrolizumab	126	QoL maintained; greater reduction in pain and improved emotional functioning with EV + P	[36]
2021	Phase III RCT	EV vs. chemotherapy	608	ORR 40.6% vs. 17.9%; median OS 12.88 vs. 8.97 months; median PFS 5.55 vs. 3.71 months	[37]
2023	Phase III RCT 24 months follow-up	EV vs. chemotherapy	608	Median OS 12.91 vs. 8.94 months; median PFS 5.55 vs. 3.71 months; ~30% alive at 24 months with EV	[7]
2023	Phase III RCT Japanese subgroup	EV vs. chemotherapy	86	ORR 34.4% vs. 21.3%; median OS 15.18 vs. 10.55 months; median PFS 6.47 vs. 5.39 months	[38]
2024	Phase III RCT	EV + pembrolizumab vs. chemotherapy	886	ORR 67.7% vs. 44.4%; CR 29.1% vs. 12.5%; median PFS 12.5 vs. 6.3 months; median OS 31.5 vs. 16.1 months	[39]
2025	Phase III RCT Subgroup analyses	EV + pembrolizumab vs. chemotherapy	886	ORR higher across subgroups; risk of progression reduced by ~55%; risk of death reduced by ~53%	[8]
2025	Phase III RCT 2.5 year follow-up	EV + pembrolizumab vs. chemotherapy	886	Median DoR ~24 months; durable responses maintained at 24 months	[40]
2025	Phase III RCT PROs	EV + pembrolizumab vs. chemotherapy	731	Improved QoL and reduced pain intensity vs. chemotherapy	[41]

## Data Availability

No new data were created or analyzed in this study. Data sharing is not applicable to this article.

## Abbreviations

The following abbreviations are used in this manuscript:

ADCs	Antibody–Drug Conjugates
ATP	Adenosine Triphosphate
CB	Clinical Benefit
cCR	Complete Clinical Response
CR	Complete Response
DAMPs	Damage-Associated Molecular Patterns
DoR	Duration of Response
EV	Enfortumab Vedotin
FDA	Food and Drug Administration
FGFR3	Fibroblast Growth Factor Receptor 3
GC	Gemcitabine-Cisplatin
HER2	Human Epidermal Growth Factor Receptor 2
HMGB1	High Mobility Group Box 1 protein
HR	Hazard Ratio
ICD	Immunogenic Cell Death
ICIs	Immune Checkpoint Inhibitors
ITT	Intention-to-Treat
laUC	Locally Advanced Urothelial Carcinoma
MDSCs	Myeloid-Derived Suppressor Cells
MIBC	Muscle-Invasive Bladder Cancer
MMAE	Monomethyl Auristatin E
mUC	Metastatic Urothelial Carcinoma
NK	Natural Killer
NMIBC	Non-Muscle-Invasive Bladder Cancer
ORR	Objective Response Rate
OS	Overall Survival
PD	Progressive Disease
PD-1	Programmed Cell Death Protein 1
PD-L1	Programmed Death-Ligand 1
PFS	Progression-Free Survival
PR	Partial Response
PRISMA	Preferred Reporting Items for Systematic Reviews and Meta-Analyses
PRO	Patient-Reported Outcome
PROSPERO	International Prospective Register of Systematic Reviews
PVRL4	Poliovirus Receptor-Related 4
QOL	Quality of Life
RCTs	Randomized Controlled Trials
RWE	Real-World Evidence
SD	Stable Disease
TILs	Tumor-Infiltrating Lymphocytes
TMB	Tumor Mutational Burden
TME	Tumor Microenvironment
TRAEs	Treatment-Related Adverse Events
Tregs	Regulatory T-cells
UC	Urothelial Carcinoma
UTUC	Upper Tract Urothelial Carcinoma
